# Best Practices in Organizing Digital Transformation: Qualitative Case Study in Dutch Hospital Care

**DOI:** 10.2196/63576

**Published:** 2025-05-08

**Authors:** Tanja Schiffelers, Kaya Kapteijns, Laura Hochstenbach, Bas Kietselaer, Esther Talboom-Kamp, Marieke Spreeuwenberg

**Affiliations:** 1 Digital Care Transformation, Zuyderland Medical Centre Sittard-Geleen The Netherlands; 2 Maastricht University Maastricht The Netherlands; 3 Department of Health Services Research, Care and Public Health Research Institute (CAPHRI) Faculty of Health Medicine and Life Sciences Maastricht University Maastricht The Netherlands; 4 Department of Cardiovascular Disease Mayo Clinic Rochester, MN United States; 5 National eHealth Living Lab Leiden University Medical Center Leiden The Netherlands

**Keywords:** digital transformation, sustainability, hospitals, barriers and facilitators, digital health literacy, implementation strategies, qualitative research, accessibility, affordability, quality, data-driven care

## Abstract

**Background:**

The health care sector faces increasing pressure, with demand outpacing supply and multiple challenges in accessibility, affordability, and quality. The current organization of health care systems is unsustainable—exacerbated by labor shortages and escalating expenditures in Europe, particularly the Netherlands. To address these issues, hospitals are increasingly adopting digital transformation strategies. This digital transformation involves the systematic implementation of digital technologies and processes. To achieve high-quality hybrid care, hospitals must integrate digital health care seamlessly into existing workflows. However, there is no definitive strategy for implementing these transformations.

**Objective:**

This study examines how Dutch hospitals organize their digital transformation, the strategies they employ, and the best practices they follow, to provide evidence-based recommendations for hospitals embarking on similar initiatives.

**Methods:**

A qualitative multicase study was conducted using purposive sampling. A total of 11 Dutch hospitals were invited, and 8 participated. Professionals—project or program managers of digital care, or advisors in policy, management, strategy, or related positions—from these hospitals took part in semistructured interviews. Topics included digital transformation strategies, organizational structures, barriers and facilitators, and lessons learned. All interviews were recorded, transcribed verbatim, and analyzed using directed content analysis.

**Results:**

Although hospitals organize their digital transformation in different ways and with different teams or departments, they encounter similar facilitators and barriers. Inspired by the Consolidated Framework for Implementation Research, the ExpandNet Scaling Up framework, and the Hybrid Health Care Quality Assessment, these factors were grouped into the following categories: the structure of the digital program, cultural factors within the organization, financial factors (internal or external), political factors (internal or external), patient needs, resources and skills, and technical factors.

**Conclusions:**

Despite variations in implementation, hospitals share key challenges and enablers in digital transformation. Common factors—such as organizational culture, financial resources, and technical infrastructure—may serve as foundational elements for effective digital transformation in hospital care.

## Introduction

### Background

Health care is under pressure: demand is increasing faster than supply. Hospitals around the world face multiple challenges related to the accessibility, affordability, and quality of health care systems—particularly in the Netherlands and other European countries [1,2]. The way current health care systems are organized is unsustainable, in terms of both labor capacity and financial viability [3]. Although specific projections for hospital care are lacking, total health care spending as a percentage of gross domestic product is expected to continue rising in the coming years, partly due to factors such as aging and technological developments. The Netherlands Bureau for Economic Policy Analysis (CPB) estimates that collective health care spending could increase from 11.2% of the gross domestic product in 2021 to over 18% by 2060 [4,5]. To create and maintain a sustainable health care system, structural changes are necessary.

Given the lack of staff, the increasing demand for health care, and technological developments, hospitals are increasingly utilizing the opportunities that technology and data-driven care offer within the health care process. Digitalization has affected—and is expected to continue affecting—various aspects of health care systems, including culture, organizations, overall outcomes, the roles of health care professionals, and treatment options [6]. It is defined as the “use of digital technologies in the context of the production and delivery of a product or service” [7]. Digital transformation refers to the systematic and deliberate integration of digital technologies and processes into health care systems. It involves not only the adoption of new digital tools, but also the reorganization of care delivery, infrastructure, and workflows to optimize hybrid care models. This transformation includes innovations such as electronic health records, decision support systems, and remote patient monitoring, among others [7,8].

While research on digital transformation has predominantly focused on technologies or differences between hospitals and patient groups, recent studies highlight the need for further investigation into strategies, stakeholder involvement, and overall impact. Achieving sustainable digital health care requires seamless integration into existing processes and adaptation to deliver high-quality hybrid care. However, there is no standardized approach to implementing digital transformation. Key strategic questions remain, such as “How should a hospital define and implement its digital strategy?” “Should responsibilities be organized centrally or locally?” and “What preconditions must be established to facilitate effective transformation [9]?”

Beyond implementation, hospitals must structure their digital initiatives across the organization, ensuring alignment with broader health care objectives. Various frameworks offer guidance in this process, including the Consolidated Framework for Implementation Research (CFIR), which addresses general implementation strategies; the ExpandNet Scaling Up framework, which focuses on scaling innovations in health care; and the Hybrid Health Care Quality Assessment (HHQA) model, a quality management and self-assessment tool [10-12].

Although eHealth technologies offer promising solutions to address health care challenges—such as improving patient self-management, disease prevention, diagnostics, monitoring, and treatment—they also present significant implementation challenges. A critical issue is digital health literacy within the general population. Disparities in digital competence can create a digital divide, preventing certain patient groups from benefiting from digital health care innovations. This is particularly concerning, as individuals with low health literacy are often those most in need of effective health care interventions. Hospitals must account for these disparities in their digital transformation strategies to ensure equitable access and usability [13-15].

### Objective

Exploring the impact of digital transformation is crucial in alleviating pressure on the health care system. By understanding how Dutch hospitals organize their digital transformation, this study aims to offer valuable insights that can help other hospitals in the Netherlands and beyond shape and improve their own digital transformation efforts. The findings will provide recommendations for the effective organization of digital transformation journeys, along with the identification of common barriers and facilitators encountered in the process. This study seeks to answer the following questions:

- How do Dutch hospitals currently organize their digital transformation?
- What are the barriers and facilitators in organizing digital transformation?
- How do Dutch hospitals perceive and experience the organization of their digital transformation?
- What best practices can be identified from these hospitals’ digital transformation efforts?

## Methods

### Study Design

We conducted a qualitative multicase study, which provides a holistic and detailed description of the experiences of hospitals with their digital transformation.

### Setting

This study took place in the Netherlands, where the health care sector accounts for 15% of total employment and constitutes a significant part of the economy. The country has 69 nonprofit hospital organizations, including 8 university medical centers, operating across 113 hospital locations and outpatient clinics. Various hospital associations contribute to shaping health care policy and driving innovation.

The Dutch Association of Hospitals (NVZ) represents general hospitals, rehabilitation centers, and specialized institutions, with a strong focus on digital transformation to address accessibility, affordability, and workforce challenges. The Association of Top Clinical Hospitals (STZ) comprises 27 leading hospitals dedicated to top clinical care, research, and innovation. Additionally, mProve and Santeon are networks of 7 STZ hospitals each, collaborating on future health care solutions.

Given the strong collaboration within the research group and the nationwide presence of mProve hospitals, this study primarily focused on their digital transformation strategies. Additionally, 4 other hospitals—1 university medical center, 2 categorical NVZ hospitals, and 1 NVZ/STZ hospital—were invited based on established collaborations [15].

### Population

A total of 11 hospitals in the Netherlands were invited to participate via email. This study specifically targeted participants that are project or program managers active in the digital transformation within those hospitals, or participants that work in a policy, strategy, management, or similar position directed at the digital care transformation in those hospitals.

The following inclusion criteria were applied for selecting interviewees within the hospitals: the hospital had to be located in the Netherlands; participants were required to have been employed at the hospital for a minimum of 3 months to ensure sufficient experience; and participants had to hold a position related to policy, management, or strategy in digital transformation within their hospital. Participants were excluded if they did not complete the informed consent before the study or if their hospital was not engaged in specific digital transformation programs or policies [16,17].

The email invitation included the informed consent forms, which explained the purpose of the research, the expectations for participants, how personal data would be handled with respect to confidentiality, and what would be done with the results. The informed consent also included an agreement to record the interview. Participants were given a 14-day deadline to confirm their participation. If no response was received within 7 days, a reminder was sent to improve recruitment success. A maximum of 3 participants per hospital was set to reduce the risk of information overload and to facilitate well-organized interviews [18,19].

### Data Collection

Data collection through individual semistructured interviews took place between April and June 2023. Before the interviews, participants were asked to share relevant documents, such as vision or policy documents related to digital transformation within their hospitals. Information from these documents concerning digital strategy was organized according to the interview topics—digital transformation strategies, organizational structures, barriers and facilitators, and lessons learned—and was used by the researchers as background information during the interviews. As all participants were native Dutch speakers, the interviews were conducted in Dutch via the Microsoft Teams platform. All interviews were recorded and transcribed verbatim. Summaries were shared with participants for member checking. Data collection continued until data saturation was reached, meaning no new themes or insights emerged from subsequent interviews.

### Data Analysis

We analyzed the data using the content analysis method, which focuses on extracting depth and detail from information gathered through open-ended data collection techniques such as semistructured interviews. The entire data analysis was conducted in ATLAS.ti (Lumivero/ATLAS.ti Scientific Software Development GmbH), a qualitative data analysis software used to organize, code, and analyze complex textual, audio, and visual data [20].

We used both deductive and inductive coding. The deductive codes were established before the analysis and were based on a combination of factors from the CFIR, the ExpandNet Scaling Up framework, and the HHQA. Inductive codes emerged during the analysis. The content analysis followed 3 phases: immersion, reduction, and interpretation [20]. In the first phase—immersion—the researcher thoroughly familiarized themselves with the data by repeatedly listening to and reading the transcripts to gain a deep understanding. Notes were taken to make sense of the data, and initial categories and themes began to emerge, which were then coded in the transcripts. In the second phase—reduction—the data were summarized, categorized, and further coded. Patterns and themes were identified to organize the data into meaningful and manageable segments, based on both deductive and inductive coding. The data were then reorganized into categories that helped address the research question. In the final phase—interpretation—the researcher analyzed the data and drew conclusions. Results from the different types of hospitals were clustered into subgroups and compared. Insights and interpretations were discussed with the research team to minimize research bias [20].

### Ethical Considerations and Ethics Approval

Ethical approval was obtained from the Medical Ethics Review Committee Zuyd (Z2022048). Participants were required to complete the informed consent form before the interview began. The informed consent clearly stated that participation was voluntary, that the data would be processed confidentially, and that participants could withdraw from the study within 14 days after data collection. To ensure the confidentiality and privacy of the participants, the data were stored in a secure file, and no personal information (eg, name, age, gender, or hospital name) was included in the transcripts or analysis.

## Results

### Characteristics of the Hospitals and Respondents

During the data collection period, the researchers approached 11 hospitals. Of these, 2 hospitals declined due to time constraints and 1 hospital did not respond. In total, 11 respondents from 8 general hospitals participated in the interviews (Table 1). The interviews lasted approximately 1 hour. All 8 hospitals that participated were general hospitals; 7 of the 8 hospitals were part of STZ and 6 were part of mProve. The hospitals varied in terms of the number of available beds and employees. The respondents held diverse roles related to digital transformation.

**Table 1:** An overview of the specific characteristics we were interested in for this study of the participating hospitals and the role of the respondents included in this study.

Hospital	STZ^a^ status	Part of mProve	Total beds, n	Total employees, n	Separate digital strategy or program	Role of respondents within digital transformation
1	Yes	Yes	>750	<5000	Yes	Digital program leader
2	Yes	Yes	>1000	>5000	Yes	Digital program director
3	Yes	Yes	>500	<5000	No	Project leader innovation Manager digital program and manager digital care center
4	No	No	<250	<2500	Yes	Manager medical information and communication technology Chief nursing information officer and coordinator digital program
5	Yes	Yes	>500	<5000	Yes	Manager care group and part-time chief medical information officer Manager of care and technology implementation
6	Yes	No	>500	<5000	No	Project leader health transformation, specific digital focus, and part-time chief medical information officer
7	Yes	Yes	>750	<5000	Yes	Manager information and care transformation Manager digital care center
8	Yes	Yes	>750	<5000	Yes	Digital program manager

^a^STZ: Association of Top Clinical Hospitals.

### How Do Hospitals Organize Their Digital Transformation?

Of the 8 hospitals, 6 had a separate strategy specifically focused on digital transformation, while 2 approached digital transformation as a means to achieve the goals outlined in their overall strategy. All hospitals had some form of team dedicated to digital transformation (n=8). The digital programs used by the hospitals varied; for example, 1 hospital had a program focused on Health care transformation in general, with 1 pillar dedicated to the digital aspect, whereas most hospitals had a comprehensive program tailored specifically to digital care transformation. Two hospitals were still refining the focus of their digital transformation programs, and 1 hospital was struggling with the operationalization of its overall digital goals. While the degree of decentralization varied across hospitals, all agreed that central support is essential to facilitate digital transformation (n=8). However, some hospitals continued to struggle with finding the right balance between decentralized operations and the central support required. Of the 8 hospitals, 6 specifically mentioned that, in addition to health care professionals, information and communication technology plays a central role in their digital transformation programs. Furthermore, 3 hospitals identified the use of a functional needs methodology for the digital transformation of care pathways. These hospitals also emphasized their focus on limiting the number of applications per functional need to prevent the proliferation of digital tools. The remaining 5 hospitals did not specifically mention using such a methodology. Table 2 provides an overview of all barriers and facilitators, organized by theme. Each theme highlights the most important or frequently mentioned barriers and facilitators as experienced by the respondents. Table 3 presents a selection of respondent quotes for each theme identified as most relevant or noteworthy.

**Table 2:** Overview of all barriers and facilitators per theme from the Consolidated Framework of Implementation Research. Each theme explains the most important or most mentioned barriers and facilitators.

Item	Facilitators	Barriers
Structure of the digital program	Involvement of health care personnel from the beginning (n=8) Setting and communicating clear goals and reporting on those goals (n=7) Invest in organizational change (n=7) Central support and facilitation (n=6) Set priorities (n=6) Multidisciplinary teams (n=5) Involvement of information and communication technology experts (n=5) Reason from the goal, not technology (n=5) Involvement of patients (n=4) Close contact between central and decentral organizations (n=4) Explain and hand over management (n=4) Consider network partners (n=4) Functional needs methodology (n=3) Including communication strategy in the program (n=3) Immediately considering upscaling (n=2)	Capacity personnel (n=6) Capacity hospital (n=5) Proliferation of applications (n=5) Lack of focus on necessary organizational change (n=3) Unclear division of labor and responsibility (n=1) Lack of consistency between long- and short-term plans (n=1) Too much synergy between digital themes (n=1)
Cultural factors within the organization	Share success (n=8) Support employees where necessary (n=6) Improve digital competencies of caregivers (n=6) Create awareness of digital strategy (n=5) Focus on behavioral change (n=5) Start with enthusiastic personnel (n=4) Give caregivers the possibilities to participate (n=4) Measure digital competencies and motivation of caregivers (n=4) Clear, open, and honest communication (expectation management) (n=4) Create a sense of urgency (n=3) Focus on motivation (n=3) Create support base (n=1) Instigate conversation (n=3) Use ambassadors (n=3)	Resistance of employees (n=8) Lack of time demotivates employees (n=6); change in the way of working (n=5) Motivation of employees (n=4) Level of digital skills of personnel (n=3)
Financial factors internal or external to the organization	Board supports the digital transformation financially (n=6) Flexible contracts with health care insurers (n=3) Allocation of funding for involving caregivers and information and communication technology experts (n=3) Transformation funds (n=3) New digital payment modules (n=1)	Lack of structural financing/reimbursement of digital care (n=8) Little flexibility and delays from health care insurers (n=7) Long and insecure return of investment (n=5) Budget cuts (n=1)
Political factors internal or external to the organization	Digital strategy that has a central place in the overall strategy (n=7) Board that supports the digital transformation (n=6) Decisiveness organization (n=5) Multidisciplinary strategy making (n=3) Policy on proven methods (n=2) National guidelines (n=1)	Board underestimates necessary organizational change (n=1) No national practical solutions (Integraal Zorg Akkoord [Integrated Care Agreement]) (n=1) Inadequate collaboration between central and decentral organizations (n=1)
Patient needs, resources, and skills	Supporting digital competencies of patients (n=6) Free choice for digital or nondigital care (n=3) Listen to and investigate where resistance comes from (n=3)	Digital skills (n=6) Willingness to participate in digital care (n=3) Adoption of digital care (n=3) Caregivers do not listen to the needs and wants of patients or do not have a correct image (n=3)
Technical factors	User-friendly technologies (n=5) Technologies that are integrated into organizations’ processes (n=5)	Links and data exchange between applications and within the network (n=7) Not integrating digital technologies into work processes (n=5) Digital technologies do not work properly (n=3) Delay because of providers (n=2) Lack of clarity on technology management (n=2)

**Table 3:** An overview of the quotes from respondents we find the most relevant or noteworthy.

Theme	Quote
Structure of the digital program	“The multidisciplinary nature of this programme means it can be successful in my experience” [hospital 8] “It’s not just about technology and digitalization, because technology gets to the patients through the healthcare providers” [hospital 1] “The digital transformation cannot be carried out without close and early involvement of ICT” [hospital 5] “Facilitate that not every department is inventing the same, we try to structure it and try to distribute the resources we have over the priorities we set and achieve efficiency where possible” [hospital 3] “And that’s the future, servicing all care pathways from those standard functional needs, no matter how crazy the care pathway” [hospital 5] “You need to focus on creating new outpatient processes, new care pathways, if you do not consider this as a major change management project, it is doomed to fail” [hospital 3] “That is also a major bottleneck, because how are you going to scale up and pace from a pilot to rolling out multiple care pathways” [hospital 2]
Cultural factors	“We know the more comfortable an employee is in using such an application, the more inclined he is to use it and encourage it in the patient as well” [hospital 8] “That you really give people time to think, but that you also pay particular attention to the obstacles they see, and allow them to name them, that goes a long way” [hospital 5] “We have just launched a major campaign for this, which starts with having the right conversation. Making sure that the people for whom it is important that they say it, feel able to say it, and find out what they need in order to say it” [hospital 1] “Good expectation management, not making false promises” [hospital 6] “The more you start to talk about it and start seeing certain successes, the bigger the group gets” [hospital 7] “The biggest challenge for me is the motivation and behavioral change of personnel” [hospital 4] “The big challenge remains how to motivate people intrinsically” [hospital 5] “They got extra work on top of that and did not see the benefits, you cannot just keep piling on, especially in that scarcity model we live in” now [hospital 3]
Financial factors	“It was a highly facilitating factor that we no longer had to submit funding per project, but that we sat down together at the same table and together decided which projects to take up” [hospital 2] “You want to make sure that people have the space to not immediately say no for time reasons” [hospital 4] “Doctors add unnecessary appointments just to get their money. Because when you are cannibalizing yourself and are constantly eating your own flesh as a hospital, then many directors will say ‘let’s quit’” [hospital 2] “What is sometimes complicated is that you often have to make some large investment in e.g. infrastructure or in buying licenses, but have some kind of lead time where you are going to find that breakeven point” [hospital 6] “It goes so slowly, really slowly. You have to spend another year negotiating with an insurer about the costs. I find that very frustrating, it slows down the pace” [hospital 2] “One of the positive factors is having a board of directors that is positively behind it, preach what you teach, you have to propagate at the top that this is the future” [hospital 6] “Our multi-year plan is focused on the digital transformation, especially on the end solution and not only on the digital part of this end solution, which I think is a very positive point” [hospital 6] “Decisiveness is really important, that we decide that we are going to do it, and then you start talking about how you are going to do it, you should not let politics take the lead in this” [hospital 7] “It (the IZA ed) is brimming with ambitions, but a lot of people believe it is limited in terms of practical solutions” [hospital 6]
Patient needs, resources, and skills	“There are so many patients that want to use digital technologies as long as you give them the right support and guidance and ask them the right questions” [hospital 8] “I often feel excluded from these kinds of pathways because in your eyes I am too old, while this is really what I need” [hospital 8]
Technical factors	“Even if another technology is better in terms of content, we would still opt for the other technology because it can be well integrated in the work processes, it is user-friendly and it has a clear overview for both patients and caregivers” [hospital 8] “For scaling up it is important that those technologies become an integral part of the processes and are not put on top of it, it should become the new process, only then you can scale up” [hospital 8] “If you don’t approach it this way, an organization will never see the benefits of the technology, because you do it on top of your regular work” [hospital 8] “And that just doesn’t work well enough in practice, and then I think ‘hello guys, you are the market leader, do something’!” [hospital 1]

### Structure of the Digital Program

All hospitals (n=8) agreed that the involvement of health care professionals is a crucial factor in the digital transformation process. Four hospitals specifically emphasized that a strong focus on professionals is both essential and a major facilitating factor. According to these respondents, when professionals support and adopt digital technologies, it significantly promotes their successful implementation. Another factor highlighted by 7 of the 8 hospitals as a facilitator was the setting and communication of clear goals, along with regular reporting and the creation of shared insight. As hospital 8 noted, it is helpful to have “continuous insight into where we are in our digitalization ambition.” Additionally, 6 hospitals mentioned that central support also acts as a key facilitator. Hospital 6 explained that centralization supports the digital transformation process. Additionally, 5 hospitals emphasized the importance of multidisciplinary teams in facilitating digital transformation, noting that the expertise of various disciplines should be integrated. Four hospitals also highlighted the importance of involving patients in the transformation process. Furthermore, 5 hospitals stressed that information and communication technology (ICT) should be an integral part of the program. The hospitals that use standardized functional needs for their digital transformation (n=3) identified this approach as a facilitating factor. According to these hospitals, it helps prevent the proliferation of applications, which is seen as beneficial. This approach supports the effective allocation of technical resources and facilitates the integration of digital technology into the hospital’s work processes.

In addition to facilitating factors, participants identified several barriers related to program structure. One commonly mentioned barrier (n=3) was the lack of focus on the organizational change required for successful digital transformation. These hospitals reported struggling with the organizational and behavioral shifts needed to support the transformation. It was emphasized that entire departments must adopt new ways of working—digital technologies should not simply be added onto existing practices. Investment in organizational change is often lacking, which is closely linked to another limiting factor: hospital capacity. Six hospitals identified limited capacity—whether in personnel, resources, expertise, or time—as a major barrier to their programs and, consequently, their digital transformation efforts. While sufficient capacity can act as a significant facilitator, during the process of scaling up, it is most often experienced as a constraint. Another commonly mentioned barrier (n=5) was the proliferation of applications, leading to a lack of oversight and insufficient integration of these tools into existing work processes.

### Cultural Factors Within the Organization

Participants also identified several cultural factors that facilitate digital transformation, most of which relate to building a strong support base within the organization. For example, by focusing on behavioral change, hospitals can enhance employees’ comfort and motivation to use digital technologies. According to all hospitals (n=8), the approach a hospital takes in fostering this support is a key facilitating factor in the success of the digital transformation. Some hospitals specifically focused on increasing the digital skills of employees (n=6), while others emphasized the importance of fostering behavioral change (n=5). Additionally, a few hospitals (n=3) highlighted that intrinsic motivation is essential for driving the digital transformation. Although hospitals noted the importance of measuring digital skills and motivation among staff and investing in these areas accordingly (n=4), they also emphasized that employees’ discomfort with using digital technologies and their lack of required skills are often underestimated. Moreover, to foster motivation, several hospitals explained that it is important to allow health care professionals to participate in shaping the digital transformation (n=7). This also highlights another facilitating factor: listening to the opinions of caregivers and addressing the barriers they encounter. Another major factor in creating a culture open to digital transformation is communication. Several facilitating factors can be identified here, such as initiating conversations about the transformation. Hospitals mentioned using various methods to do this, including plenary meetings, social media, department meetings, and even pop-up communication materials in departments. Next, a communication strategy specifically tailored to digital transformation is essential. Although all hospitals included some form of communication about the digital transformation, 3 hospitals particularly emphasized that having a dedicated communication strategy within their digital care program is crucial. This approach not only facilitates the process but also helps ensure that more employees are aware of the transformation. Another factor mentioned by 5 hospitals was ensuring that the entire hospital is aware of the digital strategy. Additionally, hospitals highlighted expectation management and honest communication as key facilitating factors (n=4). Finally, all hospitals emphasized the importance of sharing successes, with several noting that having ambassadors discuss the digital transformation and share positive outcomes is particularly effective. Communication from care professional to care professional was seen as more impactful (n=6).

Although the factors mentioned above can help motivate individuals and drive behavioral change, participants also referred to them as barriers on 4 occasions. Most of the identified barriers related to caregivers’ skills and their willingness to engage in the digital transformation. The digital skills of employees, in particular, were highlighted as a significant barrier, with 3 hospitals noting that this issue is often underestimated. Moreover, resistance from employees to participate in the digital transformation was also cited as a barrier by all hospitals. Participants identified several factors contributing to this resistance, including a natural aversion to change, a preference for physical consultations, fear that digital technologies might replace jobs, and negative financial incentives. However, most hospitals emphasized that this resistance is not typically due to a lack of willingness, but rather a result of the current challenges facing the health care system. As a result of staff shortages and high workloads, health care professionals are already under significant stress, leaving them with little time to focus on the digital transformation. Additionally, digitalization often requires more time at the outset, and when digital technologies are not properly integrated into work processes, they can add extra tasks to the regular workload. As a result, 6 hospitals highlighted the lack of time as a key reason why professionals resist digital transformation.

### Financial Factors Internal or External to the Organization

One facilitating factor that is both political and financial is the support of the hospital board for the digital transformation. Six hospitals mentioned that this support can lead to greater availability of investments and budget allocation for the digital transformation. Because of this central support, program managers and project teams do not have to focus as much on finances, as higher management handles this aspect. Another financial facilitator mentioned by 3 hospitals is having flexible contracts with health insurers, as this allows room to explore and experiment with different approaches to financing the digital transformation. Another facilitator mentioned by participants 3 times was the allocation of funds to allow health care professionals or ICT staff to invest extra time in the digital transformation. When money was available for care professionals to participate in activities related to the digital transformation, it also increased their motivation to engage. Additionally, participants cited the government’s transformation funds as a facilitator on 3 occasions. However, these hospitals emphasized that if structural funding for digital care is not secured, the removal of transformation funds could lead to a significant setback.

This brings up a major financial barrier mentioned by all hospitals: the structural financing of digital care. Because of the current financing model, there is a negative financial incentive for digital care, affecting both the board and health care professionals. This is a significant financial barrier, as the respondents noted that the current financial model does not motivate health care professionals or hospitals to advance digital transformation. As the model focuses on effort rather than results, it creates a negative incentive for digital care. Another barrier mentioned by respondents 5 times is the long and uncertain return on investment of digital technologies, which is both a financial and cultural barrier. Because of the lack of evidence, it remains unclear whether digital technologies will deliver an equivalent return on investment, both financially and in terms of care quality. This uncertainty can also demotivate personnel. Additionally, 7 hospitals mentioned that delays caused by constant negotiations with health insurers, along with the lack of flexibility from insurers, are another significant barrier.

### Political Factors Internal or External to the Organization

One of the most important facilitating political factors mentioned is board support. Six hospitals specifically noted that when the board supports digital transformation, it helps facilitate communication, finances, and overall support throughout the organization. Another factor mentioned by 7 hospitals is the need for the digital strategy to be embedded within the hospital’s overall strategy. The digital strategy should have a central place within the overall strategy and align with the hospital’s broader goals. Additionally, besides having multidisciplinary program teams, 3 hospitals noted that involving individuals with diverse backgrounds in the development of the strategy helps create a widely supported approach. Furthermore, 5 hospitals highlighted the decisiveness of the organization as a facilitating factor. Another factor mentioned by respondents twice as an important facilitator is the development of policies around effective methods. Once a method is proven to work, it should be made available to other departments to help scale-up the use of digital technologies.

One political barrier mentioned by respondents was external: national guidelines were deemed not practical enough. Additionally, an internal political barrier highlighted by 1 hospital was the managerial underestimation of the complexity of the underlying structure needed for the digital transformation. As a result, it becomes difficult to align the internal organization with the hospital’s ambition level, leading to the internal organization slowing down the digital transformation. This can also occur when the central facilitation necessary for the transformation is not in balance with the needs of the organization, which was also mentioned as a barrier.

### Patient Needs, Resources, and Skills

An important facilitating factor mentioned by 6 hospitals was supporting patients in developing their digital skills. The hospitals described several ways they tried to achieve this, such as by collaborating with partners in their network. Libraries and home-care employees helped support patients in developing their digital skills, raised awareness about the opportunities, and ensured patients felt comfortable using digital technologies. Several hospitals also established patient service points and coaching services within the hospitals. One hospital even mentioned using the patient service point as a way to assess whether their technologies were set up conveniently or if improvements could be made. This brings us to another facilitator mentioned by 3 hospitals: examining resistance from the patient’s perspective and listening to the obstacles they encounter. Hospitals mentioned that failing to do this could lead caregivers and program managers to draw incorrect conclusions, resulting in interventions that are not effective. Another facilitating factor highlighted by 3 hospitals was allowing patients to make their own choice regarding participation in digital care, focusing on those who can and want to engage with digital technologies.

Respondents also mentioned facilitators that are closely related to barriers.

One barrier highlighted by 3 hospitals was the digital skills of patients and their willingness to participate in digital care pathways. Although some hospitals argue that it is not necessary to immediately focus on these patients, others suggest that this barrier should be lowered through the facilitating factors mentioned earlier to achieve sufficient scaling of the digital transformation. Another barrier mentioned was that caregivers sometimes incorrectly assume what the patient wants, leading to the exclusion of patients who are actually willing to participate.

### Technical Factors

The last theme that emerged from the analysis was technical factors. According to 5 hospitals, an important facilitator was the user-friendliness and value of digital technology. Respondents emphasized that this is crucial for care professionals, partners in the care chain, and patients. Another facilitator identified by 5 hospitals was the extent to which a technology can be integrated into work processes. If this integration is done properly, it can facilitate digital transformation. This is why 5 hospitals mentioned that ICT should be an integral part of the process. ICT can help facilitate the transformation by indicating in advance whether something is feasible in terms of links and capacity. However, this also creates a barrier. Five hospitals noted that failing to integrate technologies into work processes leads to a significant barrier. In addition to the integration of digital technologies into processes, data exchange within the hospital and across the network was also identified as a barrier, mentioned by 7 respondents. For many hospitals, it is not possible to fully link certain applications, and data exchange within the network remains a challenge. This hinders hospitals and networks from fully realizing the benefits that digital technologies can offer. Furthermore, this leads to resistance, as care professionals often have to perform additional actions or tasks due to insufficient integration of digital technologies. This is one of the reasons why the proliferation of applications was mentioned as a barrier by 5 respondents. More applications mean more linkages to be made. Additionally, when a hospital uses too many applications, it becomes difficult to focus, and there’s insufficient capacity to manage everything effectively. Another barrier mentioned by interviewees 4 times is delays caused by technology providers or issues with the technologies themselves. Some providers are unable to ensure that the technology functions properly or can be sufficiently integrated with other systems.

## Discussion

### Principal Findings

This study aims to gain insights into how a selection of Dutch hospitals organizes their digital transformations, the strategies they use, their experiences, and the lessons learned. Although hospitals organize their digital transformations in various ways and with different teams or departments, they all encounter similar facilitators and barriers. Inspired by the CFIR, the ExpandNet Scaling Up framework, and the HHQA, these factors can be categorized into the following areas: the structure of the digital program, cultural factors within the organization, financial factors (both internal and external to the organization), political factors (both internal and external to the organization), patient needs, resources and skills, and technical factors.

As previous research has indicated, the successful implementation of digital technologies requires support from clinical staff, and codevelopment can positively influence the digital transformation [21]. Central facilitation appears to be essential when organizing digital transformation. This aligns with Kotter’s change management model, as cited in Carman et al [22], which emphasizes the step of “creating a guiding coalition” in the phase of “creating a climate for change.” According to these authors, the success of a change initiative depends on the effective management of this guiding coalition and the ongoing, visible support from top leadership. Several hospitals also confirmed this, stating that the board should actively support the change process and that a dedicated team should be in place to coordinate different initiatives and allocate resources accordingly. One hospital noted that a board that underestimates the scope of the change initiative creates a barrier to effective central facilitation. However, it is important to note that central facilitation should not be confused with managers imposing the implementation on staff. One hospital emphasized this point, noting that there should be a balance between central facilitation and the decentralized ways of working within hospitals.

As mentioned earlier, scaling up digital technologies differs from routine implementation processes. To successfully enable digital transformation, these technologies must be integrated into budgets, program structures, and work processes [22]. All the hospitals acknowledged this, noting that an organizational change is essential for achieving a successful digital transformation. Despite recognizing the importance of this change, several hospitals highlighted challenges in achieving it. There are several reasons for this. First, adopting a new way of working requires a shift in behavior. Previous research has emphasized that the success of digital technologies is closely tied to their appropriate use and uptake by care professionals [23]. It is crucial for care professionals to acquire new competencies and skills to effectively work with these new digital technologies. Therefore, it is essential to arrange adequate training and education [24]. However, as 1 hospital pointed out, there is often an underestimation of the lack of digital skills among care professionals. Consequently, training and education may not receive the attention they require. Previous research has shown that caregivers who are not comfortable with digital technologies tend to view them as obstacles [25]. It is crucial, therefore, to regularly assess the resistance, skills, and motivation of health care personnel to respond effectively. Moreover, when integrating new technologies into the workflow, it is crucial to adjust responsibilities where necessary. Research has indicated that failing to do so can become a barrier to effective process integration [25]. This is also reflected in both the inner and process domains of the CFIR, as well as the “eHealth education” domain of the eHealth enhanced chronic care model (eCCM) [26]. Another factor that can hinder organizational and behavioral change is the current state of the health care system.

Health care personnel are faced with a high workload due to the increasing demand for and complexity of care, coupled with a growing shortage of health care professionals [25]. Additionally, health care expenditures continue to rise, while hospitals are simultaneously facing budget cuts imposed by the government [27]. This results in limited hospital capacity, leaving health care personnel with insufficient time to focus on the digital transformation. As a consequence, this creates barriers to the organizational and behavioral changes needed. The concept of absorptive capacity for change in an organization [28] refers to both the resources available for the change and the relative priority that individuals assign to the change. Hospitals mentioned that, due to current health care challenges, caregivers often have other priorities that take precedence. Another potential barrier identified is the lack of focus on patients. Digital technologies aim to empower patients, transforming them into active decision makers in their own care process [28]. As a result, patients become not only “partial employees” but also “coproducers of services.” As a result, it can be argued that hospitals should directly involve patients in the change process, ensuring that their digital skills, motivation, and experiences are considered just as much as those of health care personnel. This approach is also emphasized in the eCCM, which incorporates eHealth education and training to boost patients’ self-efficacy and confidence in using digital tools [25]. Moreover, the adapted CFIR includes a sixth domain, “patient needs and resources,” as research has confirmed the importance of focusing on patients in patient-centered transformations [10].

The methods and structures underlying this process are closely tied to the organizational change. Hospitals specifically mentioned that when digitizing a care path, they offer various digital functional needs to be integrated within that path. This approach is seen as a significant facilitator, as it enables a more integrated and cohesive approach to the digital care transformation. Moreover, this structured approach can help prevent the proliferation of applications, ensuring a more streamlined digital transformation process. However, hospitals that incorporated digital functional needs into their organizational change process found it to be highly facilitating. It was noted that while offering a broader range of choices for decision-making can be beneficial, it can also complicate the implementation process, as a higher number of options often increases the difficulty of execution [29]. One could argue that functional needs provide a structured framework that reduces complexity, thereby facilitating the digital transformation process. Additionally, the CFIR construct “process” highlights the importance of developing a clear and defined method for implementing an intervention. This aligns with the product-based planning approach [30], which can be instrumental in organizing and streamlining the digital transformation effort.

All hospitals emphasized that involving various stakeholders is a key facilitating factor in the digital transformation process. Some hospitals highlighted the central role of ICT within the program team, while others focused on the importance of health care professionals’ involvement. A few hospitals stressed the need for a balanced approach, ensuring both ICT and health care professionals are equally engaged in the transformation. Although the hospitals had varying opinions on which stakeholders to involve and when during the process, they all included similar stakeholders at some point. Previous research has shown that when ICT personnel are not involved from the outset, it can slow down the implementation process and lead to a range of issues [31]. For instance, compatibility assessments were not conducted thoroughly until errors and instabilities arose, due to the lack of early involvement of ICT personnel [32]. This underscores why several hospitals opt to give ICT personnel a central role in the program. According to them, this approach facilitates better resource allocation and allows for the early assessment of potential technology integration within hospital processes. Moreover, research has shown that cocreating with health care professionals can enhance the user-friendliness and acceptability of new digital health technologies [33]. Therefore, it can be argued that the early involvement of both ICT personnel and health care professionals, along with other relevant stakeholders, is essential for successfully organizing the digital transformation process. Santarsiero et al [32] also highlighted this in their research, emphasizing that the involvement of all the organization’s stakeholders is critical for a successful digital transformation process.

Another notable factor in the results is how hospitals communicate about their digital transformation. While all hospitals engaged in various forms of communication, only a few mentioned considering communication as a central part of their program and developing or using a specific communication strategy. Previous research emphasizes the importance of communication in conveying the vision of digital transformation [30]. This is crucial because digital transformation involves organizational change, and effective change management is essential for successfully implementing such transformation.

Reimbursement of digital technologies is another critical factor for achieving a successful digital transformation in the long run. Respondents explained that the current financial model in the Netherlands focuses on effort rather than results, which creates a negative incentive for adopting digital solutions. Previous research has highlighted the importance of establishing financial mechanisms that support the organizational changes required to adopt digital technologies [34]. Currently, there are negative incentives for health care professionals and hospitals to engage in digital transformation, as it may adversely affect their reimbursement and income. Previous research on the implementation of patient portals in hospital settings confirms the barriers identified in our study, underscoring the need to reduce or eliminate these barriers for successful digital technology adoption. Furthermore, research has emphasized that redesigning business models and incentives can help overcome cost-related barriers [35]. Therefore, one could argue that a potential solution might be to create a financial model that focuses on incentives based on patient outcomes rather than activities. Ross et al [28] also support this argument in their research. Unfortunately, this is not a lesson that hospitals themselves can easily implement, as they do not have the authority to adjust the current financial models. Up until now, only 1 hospital in the Netherlands has succeeded in approaching health care financing differently, taking the first step toward value-driven contracting with health insurers [35]. However, all the hospitals emphasized the importance of removing the current financial barriers to achieve a successful digital transformation. While transformation funds do provide short-term financial incentives, they are not enough to sustain long-term progress. Nevertheless, based on the results, it can be argued that if the structure of the financial model remains unchanged, the digital transformation will not be sustainable in the long term. Support from health care professionals is crucial for the success and sustainability of the transformation, and without addressing these financial barriers, it will be difficult to maintain this support.

### Strengths and Limitations

This study has several strengths and limitations. First, the qualitative study design and semistructured interviews provide an in-depth and comprehensive understanding of hospital experiences with digital transformation. This approach offers flexibility, allowing for a deeper exploration of participants’ perspectives and experiences. Moreover, the use of a multicase study design enables a deeper understanding of both the similarities and differences between the hospitals, further contributing to a more comprehensive understanding of the subject matter. In addition, the use of a multicase study design strengthens the reliability of the evidence, as it allows for the identification of consistent patterns, enabling the drawing of more robust conclusions. The content analysis method used in this study also facilitated the identification of patterns, themes, and categories, resulting in meaningful and manageable data segments. Another strength of this study is the member check conducted with the respondents. This process allowed the participants to verify that the researchers accurately understood their information and to add any additional details if needed.

In this study, participants were selected based on their management roles within the organization. The term “role-related” encompasses a broad spectrum of positions, from board members to department heads. This wide range of perspectives provided valuable insights from various levels of management. However, the absence of a more detailed definition and validation of specific responsibilities and involvement may have led to variation in the depth and relevance of responses. While this approach contributed to the breadth of the data, it could also have impacted the consistency of the results. Future research could benefit from a more standardized selection procedure to ensure a consistent representation of management levels.

Although efforts were made to minimize researcher bias, there remains the possibility that it may have influenced the data collection and analysis. Additionally, while hospitals with varying characteristics were included in the study, the generalizability of the findings to the broader population of hospitals in the Netherlands may be limited. The sample size may limit the extent to which the results can be applied to other contexts. Additionally, the subjectivity inherent in semistructured interviews introduces potential limitations. These interviews rely on respondents’ subjective experiences and perceptions, which may lead to recall bias or be influenced by social desirability and other factors. Another potential limitation of this study is translation bias. One researcher collected and analyzed the data in Dutch, while the results were reported in English. As a result, translation bias may have occurred due to factors such as the interpretation of rhetorical language, cultural differences, and variations in sentence structure. Lastly, a limitation of the study is the specific period set for data collection. Not all hospitals were at the same stage of their digital transformation process. While some were actively working on scaling up, others were still in the implementation or development phase of their digital strategy. This variability may have affected the consistency and reliability of the data. Additionally, 3 hospitals could not participate due to time constraints within the given time frame.

### Recommendations for Practice

The authors offer several recommendations for hospitals in the Netherlands and other countries or health care organizations regarding the organization of their digital transformation. Ensuring there is central facilitation dedicated to overseeing the digital transformation is crucial. Hospitals can organize this central facilitation in various ways, but it is important to strike a balance between centralized management and decentralized working methods.

Furthermore, it is essential to emphasize the organizational and behavioral changes required for a successful digital transformation. The board of directors plays a critical role in driving and supporting the necessary changes. As hospitals implement and scale-up new initiatives, they should immediately assess how these initiatives integrate into the organization’s existing processes. If needed, responsibilities should be adjusted, and new duties should be distributed among employees to ensure smooth adoption.

Moreover, hospitals should engage professionals and other relevant stakeholders early on when implementing or scaling up initiatives. Special attention should be given to their digital skills, as well as those of the patients. To facilitate behavioral change, both groups should receive comprehensive support to ensure they are confident and capable of using digital technologies.

Finally, hospitals should develop a communication strategy focused on the digital transformation and ensure it occupies a central role within the overall transformation program. This approach can help overcome several barriers and facilitate both organizational and behavioral change. It is particularly beneficial if the board is actively involved in this communication, supporting and promoting the digital transformation, and utilizing ambassadors to help advocate for the changes.

### Implications and Recommendations for Further Research

The authors recommend several avenues for future research. One key question that remains unanswered is which of the factors identified in this study is most impactful. To address this, we suggest conducting an in-depth study in 1 hospital, validating the HHQA model in a real-world hospital setting. This would help create a comprehensive toolkit for other hospitals, providing them with actionable insights on how to effectively organize and manage their digital transformation.

In addition to focusing on specific factors, the authors also recommend conducting research centered around a key stakeholder: the patient. Understanding how patients experience digital transformation and identifying their needs will provide valuable insights. This research could lead to a more efficient and effective implementation of eHealth technologies, ensuring they are better tailored to meet the needs of patients.

Another important consideration for future research is to explore the impact of communication strategies on the success of digital transformation initiatives. It would be valuable to investigate the effectiveness of different communication methods and channels in raising awareness, stimulating motivation, and fostering the adoption of digital technologies. Another recommendation is to conduct a longitudinal study to gain deeper insights into hospital experiences at different stages of the digital transformation process. This would also help create a better understanding of the long-term effects and sustainability of various digital transformation efforts.

### Conclusions

This study aimed to explore how hospitals currently organize their digital transformation, their experiences, and the barriers and facilitators they encounter in this process. Overall, it can be concluded that while hospitals employ various approaches to organizing their digital transformation, there are also significant similarities in the methods used across different institutions. Based on the findings and prior research, we recommend establishing a central facilitation for the digital transformation process. It is crucial to focus on the organizational and behavioral changes associated with digital transformation. Hospitals can support this by assessing the resistance, digital skills, and motivation of both employees and patients, and responding accordingly to facilitate smoother transitions. Additionally, leveraging digital functional needs in the digital transformation can be beneficial, as it provides a structured approach that can reduce complexity and enhance integration. Moreover, engaging relevant stakeholders with expertise from the outset, as well as developing a communication strategy, can greatly facilitate the digital transformation process. However, while these factors can support the transformation, it is crucial to adjust the structure of the financial health care model in the Netherlands to better accommodate the use of digital technologies, ensuring sustainability and long-term success. Lastly, support from the board is essential to drive the necessary changes, and investing in the development of digital skills for both employees and patients is crucial. Without these efforts, the digital transformation is unlikely to reach its full potential.

## Abbreviations

CFIR: Consolidated Framework for Implementation Research
CPB: Netherlands Bureau for Economic Policy Analysis
eCCM: eHealth enhanced chronic care model
HHQA: Hybrid Health Care Quality Assessment
ICT: information and communication technology
IZA: integraal zorg akkoord (integrated care agreement)
NVZ: Dutch Association of Hospitals
STZ: Association of Top Clinical Hospitals

## Data Availability

All transcripts generated or analyzed during this study are available from the corresponding author upon reasonable request.
